# Whey Protein Supplementation Effects on Body Composition, Performance, and Blood Biomarkers During Army Initial Entry Training

**DOI:** 10.3389/fnut.2022.807928

**Published:** 2022-03-07

**Authors:** Jeremy S. McAdam, Kaitlin D. Lyons, Darren T. Beck, Cody T. Haun, Matthew A. Romero, Petey W. Mumford, Paul A. Roberson, Kaelin C. Young, Keith R. Lohse, Michael D. Roberts, JoEllen M. Sefton

**Affiliations:** ^1^School of Kinesiology, Warrior Research Center, Auburn University, Auburn, AL, United States; ^2^Healthspan, Resilience, and Performance Research, Florida Institute for Human and Machine Cognition, Pensacola, FL, United States; ^3^Molecular and Applied Sciences Laboratory, School of Kinesiology, Auburn University, Auburn, AL, United States; ^4^Department of Cell Biology and Physiology, Edward Via College of Osteopathic Medicine (Auburn Campus), Auburn, AL, United States; ^5^Fitomics, LLC, Pelham, AL, United States; ^6^Neurorehabilitation Informatics Lab, Department of Health, Kinesiology, and Recreation, University of Utah, Salt Lake City, UT, United States

**Keywords:** human performance, military, supplementation, fat free mass, fat mass

## Abstract

This study assesses if a lower dose of whey protein can provide similar benefits to those shown in previous work supplementing Army Initial Entry Training (IET) Soldiers with two servings of whey protein (WP) per day. Eighty-one soldiers consumed one WP or a calorie matched carbohydrate (CHO) serving/day during IET (WP: *n* = 39, height = 173 ± 8 cm, body mass = 76.8 ± 12.8 kg, age = 21 ± 3 years; CHO: *n* = 42, 175 ± 8 cm, 77.8 ± 15.3 kg, 23 ± 4 years). Physical performance (push-ups, sit-ups, and a two-mile run) was assessed during weeks two and eight. All other measures (dietary intake, body composition, blood biomarkers) at weeks one and nine. There was a significant group difference for fat mass (*p* = 0.044) as WP lost 2.1 ± 2.9 kg and had a moderate effect size (Cohen's d: −0.24), whereas the CHO group lost 0.9 ± 2.5 kg and had only a small effect size (d: −0.1). There was no significant group-by-time interaction on fat-free mass (*p* = 0.069). WP gained 1.2 ± 2.4 (d: 0.1) and CHO gained 0.1 ± 3 (d: 0) kg of FFM on average. There was a significant group by week 1-fat free mass interaction (*p* = 0.003) indicating individuals with higher initial fat-free mass benefitted more from WP. There were no group differences for push-up (*p* = 0.514), sit-up (*p* = 0.429) or run (p = 0.313) performance. For all biomarkers there was a significant effect of time as testosterone (*p* < 0.01), testosterone to cortisol ratio (*p* = 0.39), and IGF-1 (*p* < 0.01) increased across training and cortisol (*p* = 0.04) and IL-6 (*p* < 0.01) decreased. There were no differences in groups across IET for any of the biomarkers. We conclude one WP serving is beneficial for FM and for FFM in soldiers with high baseline FFM but may not significantly alter biomarker response or physical performance of IET soldiers who have high relative dietary protein intakes.

## Introduction

Improving physical fitness is key for the success of military personnel due to the strenuous nature of daily soldiering tasks (1, 2). Initial Entry Training (IET) is a physically and mentally rigorous training environment designed to prepare soldiers to perform their duties. Past research indicates that US Army IET soldiers participate in at least 6–7 h of daily physical activity, ranging from low to very vigorous intensity (3, 4). Recent research suggests Army IET soldiers may be inadequately fueled to respond optimally to large volumes of training (3). IET soldiers consume between 1,900-and 2,600 calories per day (3, 5). However, they are estimated to expend over 3,200 calories per day, resulting in a negative energy balance (3). This may have negative effects on performance and body composition (6). Research in US Army (5) and Marine (7) IET revealed that IET soldiers lost 1–3 kg of fat-free mass (FFM) on average across training, with only 36% of male Army IET soldiers gaining FFM during training (5). Losses in FFM may lead to decrements in physical performance for IET soldiers as has been found in non-IET in military training in the US and Australia (8, 9).

Serum biomarkers are one method of assessing responses to military training. Testosterone and Insulin-like Growth Factor 1 (IGF-1) are anabolic hormones that are positively related to body composition and performance due to their ability to stimulate anabolic mechanisms such as increased muscle protein synthesis (10, 11). While intense military training has been shown to reduce serum testosterone and IGF-1 (10), these reductions can be nutritionally modulated (12). Serum testosterone and IGF-1 are decreased during periods of negative energy balance across military training. These decreases can be restored to baseline levels when adequate nutritional provision is provided (12). Military training has also been shown to increase serum cortisol levels, a hormone that results in skeletal muscle catabolism (9, 12). The balance between anabolic and catabolic hormones is thought to be important for the promotion of muscle remodeling. Imbalances in the testosterone: cortisol (T:C) ratio, whether it is caused by decreases in testosterone or increases in cortisol, have been shown to be associated with reductions in performance (13). Studies in Army Rangers (12), Australian basic training (14), and United Kingdom section commanders' battle course (15) all report that military training results in elevated cortisol levels and a reduction in the T:C ratio. Military training has also been reported to increase serum cytokine concentrations, such as interleukin-6 (IL-6), that stimulates the inflammatory response to muscle damage and pathogens (16–19). Studies in Norway and France show that military training can lead to increases in IL-6 acutely (four days) and chronically (four weeks) (18, 19). Chronically elevated levels of IL-6 have been related to overtraining and may represent inadequate recovery (20).

Nutritional supplementation may have an important influence on physical and hormonal responses to military training. One United Kingdom study (9) reported the addition of a protein-based supplement negated the decrease in performance and FFM during 8 weeks of training. Our previous work in IET soldiers revealed supplementation with either a higher dose (two servings per day) of whey protein or a calorie-matched carbohydrate (CHO) resulted in a higher percentage of participants gaining FFM across IET in comparison to a previous investigation of non-supplemented IET soldiers (5, 21). Additionally, two servings per day of WP resulted in significantly higher push-up performance and potentiated reductions in fat mass (FM) in comparison to CHO (21). WP with small amounts of casein has been shown to increase IGF-1 and muscle mass in individuals involved in strength training (22). Another study (23) found that 6 months of protein supplementation resulted in increases in serum IGF-1 levels in individuals involved in concurrent strength and endurance training. The effects of protein supplementation on IGF-1 levels are thought to be mediated by an increased supply of amino acids that stimulate IGF-1 gene transcription in skeletal muscle (22). WP has also been reported to increase serum testosterone in comparison to soy, as well as to reduce serum cortisol levels in response to resistance training compared to soy and carbohydrate (CHO) supplementation (24). Collectively, these studies suggest that WP may be beneficial for improving the hormonal environment required to support advantageous physical performance and physiologic responses to IET.

The goal of the current study is to build upon our previous research examining the impact of WP supplementation on IET soldiers. Here we examined if one WP serving per day was more beneficial than CHO on performance, body composition, and serum-biomarker responses. If one WP serving per day provides similar benefits to those demonstrated with two WP servings per day, it would reduce preparation and distribution time, as well as supplementation costs for the military. Based on our prior data, we hypothesized WP would be beneficial for push-up performance and body composition. Additionally, we hypothesized that WP would be more beneficial than CHO for improvements in the T:C ratio and IGF-1 responses to training due to improvements in the anabolic status of the body as well as reductions in cortisol and IL-6 which may indicate improved recovery during IET.

## Methods

### Study Design and Population

This was a double-blind, placebo-controlled, 2 x 2 (Group x Time) factorial-repeated measures design. The Auburn University Institutional Review Board, and the Director, Research, and Analysis Directorate Army Center approved the study procedures for Initial Military Training. Potential participants were given a description of the study. Those wishing to participate gave written consent and were enrolled in the study. Participants were cleared for military training and were apparently healthy 19–35-year-old men engaged in Army IET. All IET soldiers are required to live in barracks under the continual supervision of drill sergeants throughout the duration of IET. Daily schedules are highly regimented according to Army regulations from the time IET soldiers wake until time for bed. Daily physical fitness and occupational training events are performed in groups led by Army leadership. Daily activities consisted of morning group physical fitness (bodyweight resistance training, endurance training, general flexibility, and calisthenics) followed by soldier training tasks (ruck marching, obstacle course, land navigation, battle tactics training, field training exercises, etc.). All soldiers in the unit completed the same tasks for the same duration each week. All soldiers consumed food from the same menu, and meals were consumed from the dining facility or from pre-packaged meals ready to eat. Participants were free from musculoskeletal injury (MSI), allergies to milk or whey protein, and had not taken supplements within the past 3 months. In total 95 participants agreed to participate in the study, 81 participants completed the study (WP: *n* = 39, height = 173 ± 8 cm, body mass = 76.8 ± 12.8 kg, age = 21 ± 3 years old; CHO: *n* = 42, 175 ± 8 cm, 77.8 ± 15.3 kg, 23 ± 4 years old). A total of 14 participants were removed from the analysis due to prior supplementation (four participants), lack of adherence to supplementation (five participants), discontinued IET (four participants), and withdrawal of participation in the study (one participant).

Participants were supplemented with either one whey protein (Power Crunch® ProtoWhey® (BioNutritional Research Group; Irvine, CA, USA) as agglomerated, partially hydrolyzed (12.5% degree of hydrolysis) 80% whey protein concentrate (Hilmar® 8360; Hilmar Ingredients, Hilmar, CA USA) or calorie-matched CHO supplement per day. Supplement manufacturing and formulation have been described previously (21). Briefly, all supplements were manufactured at JW Nutritional, LLC (Allen, TX, USA), a United States Food and Drug Administration cGMP-compliant facility independently audited and pre-qualified by Obvium^*^Q, LLC (Phoenix, AZ, USA), a GMP regulatory compliance firm. Personnel at JW Nutritional, LLC and C.M.L. (Lockwood, LLC; Draper, UT, USA) formulated supplements to match for taste. These entities also maintained blinding of groups, and each supplement was assigned a randomly generated item number. The research team and participants were blinded to the contents of the packets until data collection was completed. Manufacturing batch records for production of each of the supplements were reviewed by a trained, independent expert in dietary supplement quality control, taste, and assurance (C.M.L.) before approval for use within the present study. The nutritional profile and amino acid content of both supplements were third-party tested by Covance Laboratories, Inc. (Madison, WI, USA) to verify the identity, purity, potency, and composition of the packets. The nutritional profile is described below in Table 1. In order to minimize interference in the IET training schedule, each week, all supplement packs were provided to the drill sergeants for their respective platoon. The drill sergeants then provided the supplements to the IET soldiers who were instructed to consume the shakes before bedtime. To assess adherence, the research team checked the boxes that were delivered to ensure distribution and asked the IET soldiers to report the number of shakes missed during the study.

**Table 1:** Supplement nutrition information.

**Macronutrient**	**WP Supplement**	**CHO Supplement**
Energy (kcal)	293	291
Protein (g)	38.6	0.5
Carbohydrate (g)	19	63.4
Fat (g)	7.5	3.9
Essential AA (g)	20.1	0.1
BCAA (g)	9.5	0.0

*WP, Whey Protein supplement; CHO, Carbohydrate supplement; Kcal, Kilocalories; g: grams; AA, Amino Acids; BCAA, Branched Chain Amino Acids*.

### Measures

**Figure 1:**
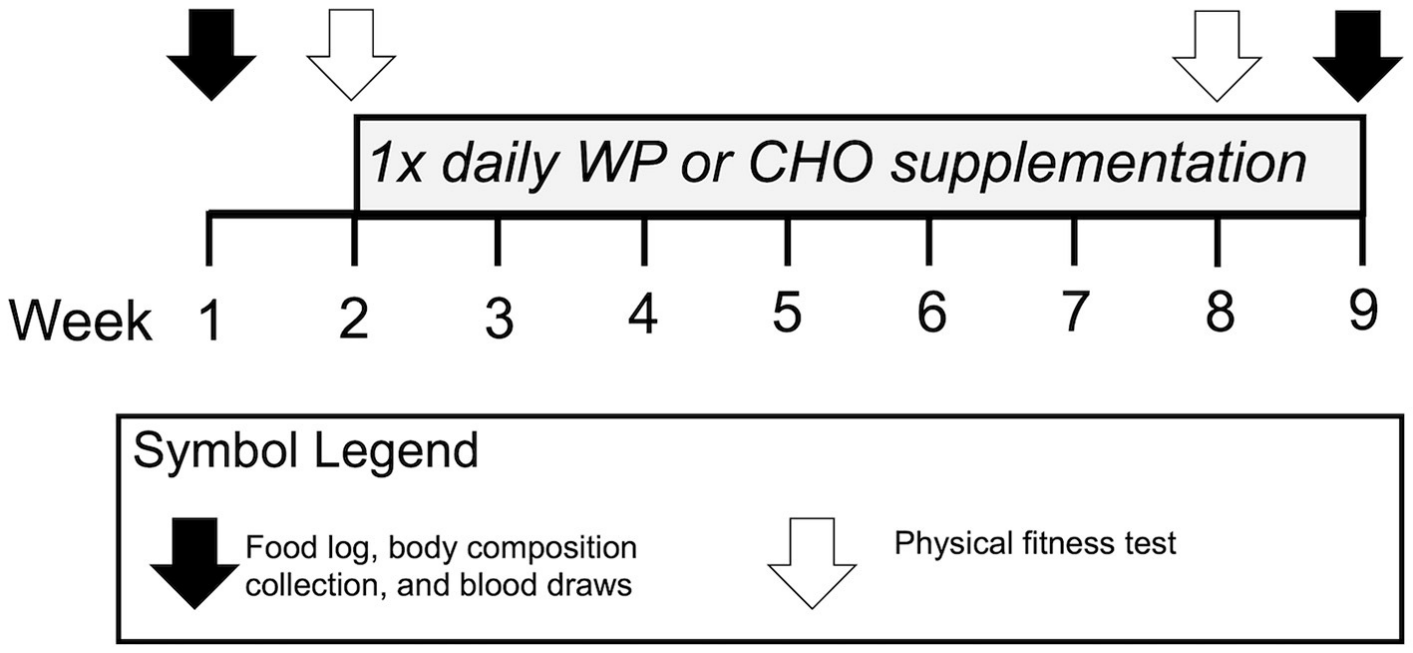
Study timeline and measures. WP, Whey protein supplement group; CHO, Carbohydrate supplement group; 1x, Once daily.

The independent variables were supplementation group (WP or CHO) and time (week 1, week 9). Outcome variables were daily training volume, physical performance as measured by the Army Physical Fitness Test (APFT), body composition, dietary intake, serum biomarkers of anabolic status (testosterone, cortisol, IGF-1, T:C), and immune health/recovery (IL-6). Fasted blood and body composition were collected during weeks one and nine of training prior to breakfast and morning physical training. Urine-specific gravity (USG) testing was completed prior to all blood collections and body composition assessments to ensure hydration status. Participants with USG values above 1.03 were considered inadequately hydrated, given water to drink, and not allowed to proceed with testing until USG was below 1.03. Performance measures were performed during weeks two and eight of training. Figure 1 summarizes the timeline of measurements for each variable during this study.

#### Physical Activity

The methodology employed for the evaluation of physical activity levels has been previously reported in detail (3). Briefly, physical activity was estimated using Actigraph GTX monitors (Actigraph, Pensacola, FL, USA). Each week a different set of 20 participants (10 per supplement group) were asked to wear a monitor on their right hip (Actigraph protocol) for 1 week and not remove the monitor except to shower. Monitors were initialized prior to deployment and physical activity per day was estimated using Actilife software version 13.1.1 (Actigraph, Pensacola, FL, USA). Time spent in each category of physical activity was estimated using Sasaki vector magnitude 3 (VM3) (25). The following range of counts were used for each category of physical activity: Moderate = 2,690–6,166 counts/min (3–5.99 METs), Vigorous = 6,167–9,642 counts/min (6–8.99 METs), and Very Vigorous ≥ 9,642 counts/min (>9 METs) (25). All VM3 counts below 200 counts/minute were classified as sedentary (26), and the difference between sedentary cut points and moderate cut points were classified as low intensity (201–2,689 counts/min). The sampling rate was 30 Hz (27) and for the physical activity data to be considered valid, wear time as estimated by Actilife software was a minimum of 600 min (26).

#### Dietary Intake

Diet logs were completed on three days during weeks one and nine of IET. A detailed description of this process has been previously reported (3). Briefly, members of the research team obtained the menu from the dining facility IET where soldiers were required to eat at and pre-filled the diet log with options available for that meal. Immediately after the meal the research team provided the diet logs to the participants to circle the items and amounts, they ate and were available to answer any questions. Diet log data was entered into excel spreadsheets and reviewed by two researchers for accuracy. The diet data were then imported into R statistical software (28) and dietary intake calculations were completed using R Studio (29) along with the R programming packages: dplyr (30), tidyr (31), reshape2 (32), ez (33), car (34), vars (35), and ggplot2 (36). Total calorie, protein, carbohydrate, and fat intakes were calculated for each meal and day and then averaged for training weeks one and nine. Nutritional data for the dining facility foods were retrieved from the Army Joint Culinary Center of Excellence (JCOE) website and those not available on JCOE were retrieved from the US Department of Agriculture nutrition database (21). Diet logs for all three meals were required for the day to be considered valid for dietary analysis. Days in which a participant did not complete all three logs were removed from the analysis. Participants without at least two full days of diet logs each week were removed from the summary of diet logs. A total of 60 of the 81 participants who completed, had at least two full days of diet logs and were included in the dietary analysis. Diet logs during the first week were collected before the intervention period began in order to get a baseline characterization of dietary intake in the absence of supplementation (week 1-NS). We analyzed the dietary intake data in two ways: week 1-NS compared to week 9 dietary intake with supplement nutritional information included in the overall macronutrient count (week 9-SI) and excluding the macronutrient information from the overall macronutrient count (week 9-NS). Our aim was to see if there were significant changes in food that were consumed from the dining facility.

#### Body Composition

Height and body mass were assessed with participants wearing Army-issued physical training shorts, socks, and shirts using a Health-O-Meter professional scale (Model 500KL, Sunbeam products INC. Boca Raton, FL. USA) and reported in centimeters and kilograms. Body composition was assessed using an ImpediMed DF50 device (ImpediMed Ltd, Brisbane, Australia). This measure is sensitive to hydration; therefore, hydration was assessed prior to measurement through urine-specific gravity (described above). Participants were asked to lay supine for ~5 min to allow for equilibration of body fluids across intracellular and extracellular compartments prior to assessment. Measurements were taken in the supine position. Electrode placement locations on the hand and ankle were determined as per the manufacturer's recommendations. An electrode was placed on the midline of the left arm proximal to the ulnar styloid process and a distal electrode was placed on the midline 5 cm apart. Electrodes were placed on the ankle on the midline between the medial and lateral malleolus and 5 cm distal to the malleolus on the midline. All application sites were shaved to ensure optimal electrode contact. All electrode placements were performed by the same member of the research team to minimize variability. Raw output was collected from the device, and fat-free 434 mass (FFM) and fat mass (FM) were calculated using the formulas below (37):


$$\begin{array}{l} FFM=\frac{Height^{2}}{Resistance}\;*\;0.734\;+\;BW\;*\;0.116+\;Reactance\\ \;*0.096\;+\;1\;*\;0.878-4.03 \end{array}$$

$$\begin{array}{l} FM=Body\;mass-Fat\;free\;mass \end{array}$$

#### Physical Performance

The APFT was performed during weeks two and eight of the intervention. The APFT (fitness standard of record at the time of the study) consisted of a 2-min sit-up, 2-min push-up, and two-mile run. The APFT was administered by unit drill sergeants according to the standards of the US Army field manual for physical fitness training (1). Details describing APFT administration and criteria for Army standards for the proper performance of a push-up and sit-up and two-mile run have been described previously (1, 38).

#### Serum Biomarkers

Blood draws were taken from the antecubital vein *via* 21-gauge, Safety-Lok needle kits (Benton, Dickinson, and Company, Franklin Lakes, NJ, USA). Blood was collected in 10 ml serum separator vacutainer tubes (BD Vacutainer; Franklin Lakes NJ, USA) and placed on ice in a cooler (Yeti Coolers LLC, Austin TX, USA) until centrifugation the same morning of collection. Blood samples were centrifuged at 3,500 x g for 10 min at room temperature. Samples that were not fully separated were centrifuged again under the same conditions. Serum was extracted from separated blood and frozen at −80°C until analysis. Testosterone (American Laboratory Products Company, Salem, NH, USA, sensitivity: 0.022 ng/ml, CV: 2.9%), cortisol (American Laboratory Products Company, Salem, NH, USA, sensitivity: 0.4 μg/dL, CV: 4.8%), IGF-1 (American Laboratory Products Company, Salem, NH, USA, sensitivity: 0.091 ng/ml, CV: 10.5%), and IL-6 (Invitrogen, Carlsbad, CA, USA, sensitivity: 0.3 pg/ml, CV: 7.1%) were measured using ELISAs according to manufacturers' instructions. Plates were analyzed at respective wavelengths using a multispectral spectrophotometer (BioTek Eon, Winooski, VT, USA). All samples were analyzed in duplicate, and each participant's weeks 1 and 9 samples were analyzed on the same plate. All-optical densities were within the detectable range of the assays. IL-6 had four individuals whose concentrations could not be used due to being outside the normal physiologic range for the four-compartment logistic regression models and were removed from the analysis. Serum concentrations of each optical density were calculated as per manufacturer instructions using either regression or a four-parameter logistic regression.

### Statistical Analysis

For statistical analysis, ANOVA was used to compare diet and serum markers between groups and across the time of the intervention. The assumption of normality of residuals testing was completed for all variables using Shapiro-Wilks (W: Wilk's Statistic), Kolmogorov Smirnov tests, and residual QQ plots were used to visually inspect the data. Data were square-root transformed and normality was recalculated for any variable for which more than 75% of the levels were non-normally distributed. An *a priori* alpha level of 0.05 was set for the determination of significant effects. Maulchy's test of sphericity was used to evaluate equality of variance and Levene's test was used to evaluate the homogeneity of variance. If sphericity was violated a Greenhouse-Geisser correction was used. Group-by-time interactions were further evaluated using paired samples *t*-test to evaluate simple main effects of time and independent samples *t*-tests were used to evaluate the simple main effect of the group.

ANCOVA was used to evaluate performance and body composition. ANCOVA has been reported to increase sensitivity to factors specified by the study design (39). Mean centered initial values for each variable were used as the covariate in the ANCOVA model.

A mixed-design ANOVA was used to detect differences in average time spent per week training across our independent variables of the training week and supplement group. Our aim with this analysis was to determine if training volume across each intensity (low, moderate, vigorous, or very vigorous) was significantly different between supplement groups and/or across each week of IET. We employed a Tukey HSD *post-hoc* test for pairwise comparisons.

Cohen's *d* effect sizes were calculated within groups across training, as well as between groups at week 9. Effect sizes are reported as effect sizes with the associated upper and lower limits of the 95% CI. Calculations are provided below:


$$\begin{array}{l} Effect\;Size=mean\;(week9)\\ \;-mean\;(week1)/pooled\;standard\;deviation\; \end{array}$$

$$\begin{array}{l} Pooled\;standard\;deviation=Square\;root\;((SD{\left(week1\right)}^{2}\\ \;+SD\;{\left(week9\right)}^{2})/2) \end{array}$$

Testosterone violated assumption of normality at all levels (WP = W: 0.78, *p* < 0.01 week 1; W: 0.7, *p* < 0.01 week 9; CHO = W: 0.8, *p* < 0.01 week 1; W: 0.7, *p* < 0.01 week 9). Testosterone was log-transformed and re-tested for normality. Only the week 9 data were non-normally distributed, but ANOVA is robust to partial violations of normality, so we chose to proceed with the analysis. IL-6 concentrations were log transformed and normality was re-tested. Following log transformation all levels of the variable were normally distributed.

## Results

### Physical Activity

There was no statistical difference between groups for volume of training. This is indicated by a lack of significant difference between groups for light (F[1] = 0.18, *p* = 0.67), moderate (F[1] <0.01, *p* = 0.97), vigorous (F[1] = 0.03, *p* = 0.86) or very vigorous (F[1] = 0.9, *p* = 0.35) activity. There was a significant difference in light (F[2, 100] = 5.12, *p* < 0.01) and moderate (F[2, 100] = 7.02, *p* < 0.01), but not vigorous (F[2, 100] = 1.41, *p* = 0.25) or very vigorous (F[2, 100] = 2.42, *p* = 0.09) activity levels across phase of IET. For light intensity, *post-hoc* testing revealed red phase was significantly higher than blue (adj. *p* = 0.05) and white (adj. *p* = 0.01) phases. For Moderate intensity white phase was lower than red (adj. p = 0.02) and blue (adj. *p* < 0.01) phases. Total training time was only found to be significantly different between white and red phase as red phase was on average 50 min higher than white (adj. *p* = 0.01). Table 2 below summarizes the training volume during each phase.

**Table 2:** Summary of training volume per phase IET.

**Phase**	**Group**	**Light**	**Moderate**	**Vigorous**	**Very Vig**.	**Total**
Red	CHO	303 (37)	110 (23)^*^	23 (13)	5 (2)	441 (57)^*^
	WP	300 (41)	105 (23)^*^	26 (18)	6 (3)	437 (66)^*^
White	CHO	274 (36)^+^	92 (21)	19 (16)	5 (4)	391 (61)
	WP	272 (48)^+^	91 (28)	18 (11)	6 (5)	388 (78)
Blue	CHO	278 (49)^+^	110 (31)^*^	28 (40)	3 (3)	419 (93)
	WP	271 (54)^+^	122 (41)^*^	27 (33)	4 (4)	424 (106)

*Phase, Red (weeks 1–3), White (weeks 4–6), Blue (weeks 7–9); WP, Whey protein supplement group; CHO, Carbohydrate supplement group; All values are in min/day ± SD values parenthesized. Very Vig.: very vigorous*.

+ *Indicates significantly different from Red Phase*.

* *Indicates significantly different from White Phase*.

### Dietary Intake

Baseline diet was collected prior to integration of supplementation. Differences in dietary intake from the dining facility alone, between groups across IET without supplement nutritional information, and comparisons on both absolute and relative dietary intake (normalized to body weight in kg) were generated. Statistical results are listed below, and descriptive results are shown in Table 3.

**Table 3:** Summary of dietary intake across IET.

**Nutrient**	**Group**	**Units**	**Week 1-NS**	**Week 9-NS**	**Week 9-SI**
Energy	CHO	kcal/day	2,759 (585)	3,472 (697)^+^	3,763 (697)
	CHO	kcal/kg/day	37.4 (11.4)	46.7 (12.2)^+^	50.6 (12.7)
	WP	kcal/day	2,620 (626)	3,163 (765)^+^	3,456 (765)
	WP	kcal/kg/day	34.7 (10.8)	42.1 (11.9)^+^	46 (12.2)
Protein	CHO	g/day	122 (26)	163 (29)^+^	163 (29)^+^^*^
	CHO	g/kg/day	1.7 (0.5)	2.2 (0.5)^+^	2.2 (0.5)^+^^*^
	WP	g/day	118 (26)	148 (30)^+^	186 (30)^+^^*^
	WP	g/kg/day	1.6 (0.4)	2 (0.5)^+^	2.5 (0.5)^+^^*^
CARB	CHO	g/day	359 (90)	456 (105)^+^	519 (105)^+^^*^
	CHO	g/kg/day	4.9 (1.6)	6.1 (1.8)^+^	7 (1.9)^+^^*^
	WP	g/day	342 (92)	423 (114)^+^	442 (114)^+^^*^
	WP	g/kg/day	4.5 (1.5)	5.6 (1.7)^+^	5.9 (1.7)^+^^*^
Fat	CHO	g/day	95 (19)	112 (27)^+^	116 (27)
	CHO	g/kg/day	1.3 (0.4)	1.5 (0.4)^+^	1.6 (0.4)
	WP	g/day	90 (23)	99 (27)^+^	107 (27)
	WP	g/kg/day	1.2 (0.4)	1.3 (0.4)^+^	1.4 (0.4)

*Values are represented as mean (± SD). Week 1 of IET; Week nine of IET; Week 1-NS: Week 1-training, no supplement nutrition included in total; Week 9-NS: Week 9, no supplement nutrition information included; Week 9-SI: Week 9 with supplement nutrition information added to the total. WP, Whey protein supplement group; CHO, Carbohydrate supplement group*.

* *Indicates significant group difference at the respective time point*.

+ *Indicates a significant effect of time (Week 1 vs. Week 9)*.

*A priori set at p < 0.05*.

#### Dietary Intake From Meals Only

Nutritional intake with no supplementation is presented in Table 3. There were no statistical differences between groups, calories, or macronutrients consumed from the dining facility across IET. This is indicated by a lack of significant group by time interactions for absolute calorie (F[1,60] = 1.1, *p* = 0.3), protein (F[1,60] = 2.03, *p* = 0.16), fat (F[1,60] = 1.44, *p* = 0.24), carbohydrate (F[1,60] = 0.43, *p* = 0.51), cholesterol (F[1,60] = 0.54, *p* = 0.47), and sodium (F[1,60] = 0.58, *p* = 0.45) intake. This was also true when intakes were normalized to body weight. There were no significant group by time interactions for calorie (kcal/kg; F[1,60] = 0.65, *p* = 0.42), protein (g/kg; F[1,60] = 1.31, *p* = 0.26), fat (g/kg; F[1,60] = 0.89, *p* = 0.35), carbohydrate (g/kg; F[1,60] = 0.25, *p* = 0.62), cholesterol (mg/kg; F[1,60] = 0.36, *p* = 0.55), and sodium (mg/kg; F[1,60] = 0.15, *p* = 0.7).

Both groups significantly increased consumption of absolute energy (F[1,60] = 50.27, *p* < 0.01), protein (F[1,60] = 66.01, *p* < 0.01), fat (F[1,60] = 15.14, *p* < 0.01) and carbohydrate (F[1,60] = 44.39, *p* < 0.01) from week 1 to week 9. This finding remained significant when these macronutrients were normalized to body weight, as there were also main effects of time for calorie (F[1,60] = 59.58, *p* < 0.01), protein (F[1,60] = 78.06, *p* < 0.01), fat (F[1,60] = 17.29, *p* < 0.01), and carbohydrate (F[1,60] = 52.9, *p* < 0.01).

#### Dietary Intake With Supplements Included

Nutritional information with supplementation values added to the week 9 dietary intake after week one are presented in Table 3. There was a significant group by time interaction for protein, absolute (F[1,60] = 11.86, *p* < 0.01), relative (g/kg; F[1,60] = 10.89, *p* < 0.01), and carbohydrate, absolute (F[1,60] = 6.15, *p* = 0.02) and relative (g/kg; F[1,60] = 4.73, *p* = 0.03). *Post-hoc t*-tests to assess differences indicated both WP and CHO groups increased absolute protein intake across IET. The WP group increased protein intake on average 68 grams (*t* = 15.62, *p* < 0.001) and CHO increased on average 41 grams (*t* = 5.24, *p* < 0.001). There was no statistical difference between protein intake at baseline between groups (*t* = 0.6, *p* = 0.552), however there was a significant difference at week 9 (−3.04, *p* = 0.004). There was a significant increase in absolute carbohydrate intake across IET in both the WP (*t* = 6.2, *p* < 0.001) and CHO (*t* = 5.84, *p* < 0.001) groups. There was no significant difference in carbohydrate intake at baseline (*t* = 0.73, *p* = 0.469), but there were significant differences at week 9 (*t* = 2.77, *p* = 0.007). Similar findings existed when protein and carbohydrate were normalized to body weight. There were significant increases in relative protein (WP: *t* = 14.87, *p* < 0.01; CHO: *t* = 4.02, *p* < 0.01) and carbohydrate (*t* = 5.82, *p* < 0.01, *t* = 4.57, *p* < 0.01) intakes across IET in both the WP and CHO groups. For relative protein and carbohydrate intake there were no significant differences in intakes at baseline (protein-baseline: *t* = 0.82, *p* = 0.42, carbohydrate baseline: *t* = 0.86, *p* = 0.39), but there were differences at week 9 (protein-week 9: *t* = −2.06, *p* = 0.04, carbohydrate-week 9: *t* = 2.44, *p* = 0.02).

### Body Composition

A total of 81 participants were included in the analysis of body composition (BM, FM, FFM). Descriptive statistics and effect sizes are reported in Table 4. For BM, mean-centered week 1-BM was a significant predictor of week 9 BM (F = 1,420.3, *p* < 0.001). However, there were no group (F = 0.13, *p* = 0.722) or group by week 1-BM interactions (F = 0.74, *p* = 0.393).

**Table 4:** Summary of body composition and performance.

**Variable**	**Group**	**Week 1**	**Week 9**	**Mean Difference [CI]**	**Effect Size**
BM (kg)	CHO	77.8 (15.3)	76.9 (13.1)	−0.8 [−8, 6.3]	−0.04
	WP	76.8 (12.8)	75.8 (11.6)	−0.9 [−7.5, 5.7]	−0.05
FFM (kg)	CHO	61.4 (10.5)	61.5 (8.7)	0.1 [−5.8, 5.9]	0
	WP	59.5 (8.4)	60.7 (8.5)	1.2 [−3.5, 5.8]	0.1
FM (kg)	CHO	16.3 (6.6)	15.4 (5.7)	−0.9 [−5.9, 4.1]	−0.1
	WP	17.2 (7)	15.1 (5.2)	−2.1 [−7.8, 3.6]	−0.24
		**Week 2**	**Week 8**		
Run (sec)	CHO	965 (146)	849 (77)	−116 [−280, 47]	−0.7
	WP	981 (144)	870 (85)	−112 [−284, 60]	−0.67
PU (reps)	CHO	44 (17)	51 (14)	8 [−17, 33]	0.35
	WP	36 (18)	48 (15)	12 [−6, 30]	0.52
SU (reps)	CHO	51 (14)	66 (11)	15 [−0.3, 31]	0.86
	WP	44 (15)	60 (13)	16 [−9, 40]	0.8

*Raw values are represented as mean (± SD). Mean difference: the average difference across IET with 95% CIs at weeks 1 and 9 for BM, FFM, and FM and weeks 2 and 8 for Run, PU, and SU. Effect size, Cohen's D; BM, Body Mass; FFM, Fat-Free Mass in kg; FM, Fat Mass in kg; Run: two-mile run time in seconds; PU, Push-ups completed in 1 min; SU, Sit-ups completed in 1 min; kg, kilogram; sec, seconds; reps, number of repetitions completed; WP, Whey protein supplement group; CHO, Carbohydrate supplement group*.

**Figure 2:**
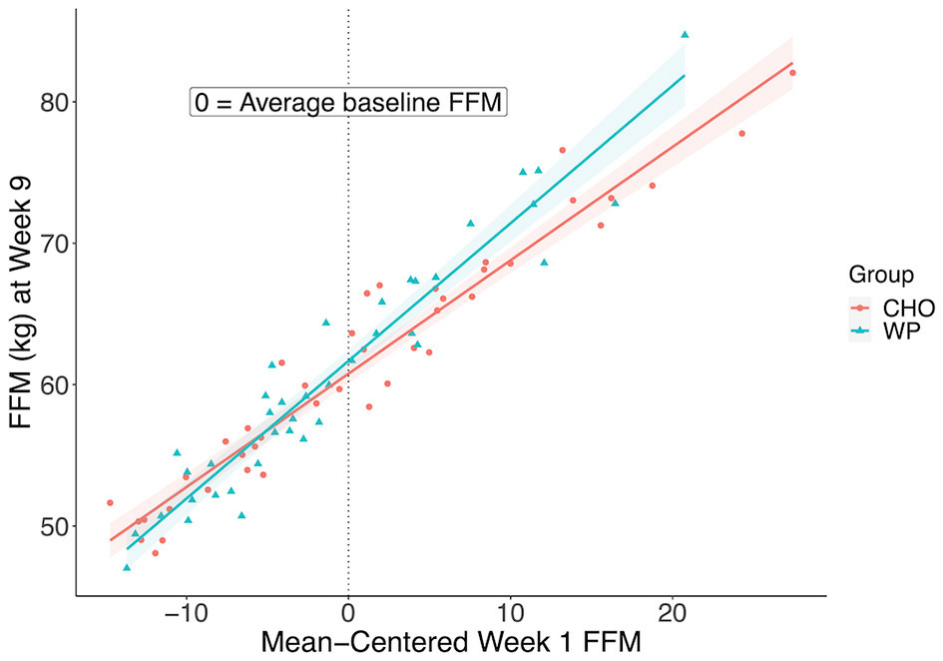
Interaction between baseline and Week 9 FFM between groups. Plot of Week 9 FFM in kilograms and mean-centered Week 1 FFM to display the relationship between groups. FFM, Fat-Free Mass; CHO, carbohydrate supplement group; WP, Whey protein supplement group.

There was a significant group by week 1-FFM interaction (F = 9.46, *p* = 0.003) on week 9 FFM and a significant interaction for week 1-FFM and group, thus the main effects could not be interpreted. Therefore, we conducted two follow-up analyses. First, linear models were fitted to the WP and CHO groups separately to investigate the influence of baseline FFM on the response to the treatment. Next, we conducted a standard group-by-time ANOVA to gain insight into the change across time in FFM between the groups. The interaction plot (Figure 2) below shows a trend in the relationship between baseline FFM and week 9 FFM depending on the group. For every 1 kg increase in baseline FFM, there was a related 0.97 kg increase in FFM at week 9 in the WP group compared to a 0.8 kg increase in FFM at week 9 in the CHO group. The coefficients from the multiple regression model (ANCOVA with the significant group by week 1-FFM interaction) were used to predict week 9-FFM as an illustration of the interaction of week 1-FFM and supplement group. If a soldier began IET at 5 kg above average in FFM the predicted week 9-FFM would be 1.76 kg higher if the soldier were in the WP group than if the soldier were in the CHO group. However, if the soldier were 5 kg below average, week 9 FFM is predicted to be only 0.04 kg higher if the IET soldier were in the WP vs. the CHO group. If these are extended to being 10 kg above or below average FFM beginning IET, the soldier who is 10 kg above average would be expected to have a week 9 FFM 2.62 kg higher if given WP vs. CHO, whereas if the soldier were 10 kg below average, the expected FFM at week 9 would be 0.81 lower if the soldier were in the WP vs. the CHO group. The group by time ANOVA trended toward significance (F=3.38, *p* = 0.07). The WP group increased FFM 1.2 kg on average and the CHO group increased by 0.1 kg on average, suggesting that WP may be beneficial for FFM response to IET. Both groups increased FFM across IET as there was a significant effect of time (F = 4, *p* = 0.05).

For FM, there was no group by week 1-FM interactions (F = 2.26, *p* = 0.137). Mean centered week 1-FM (F = 456, *p* < 0.001) and group (F = 4.18, *p* = 0.044) were significant factors for week 9 FM. WP lost 2.1 kg on average of FM across IET whereas the CHO group lost 0.9 kg.

### Physical Performance

We were only able to obtain performance data from three out of the four platoons, creating an imbalance in sample size between groups for performance metrics. In total, there were 57 participants (WP = 37, CHO = 18) data included in the analysis for push-ups and sit-ups, and 56 participants (WP = 36, CHO = 18) for run. For sit-ups, mean centered week 1-sit-up performance was a significant predictor of week 9-sit-up performance (F = 43.85, *p* < 0.001). However, there were no group (F = 0.64, *p* = 0.429) or group by week 1-sit-up interactions (F = 0.16, *p* = 0.694). For push-ups, mean centered week 1-push-up performance was a significant predictor of week 9 push-up performance (F = 96.94, *p* < 0.001). However, there were no group (F = 0.43, *p* = 0.514) or group by week 1-push-up interactions (F = 0.97, *p* = 0.33). For run performance, mean centered week 1-run performance was a significant predictor of week 9-run performance (F = 133.52, *p* < 0.001). However, there were no group (F = 1.04, *p* = 0.313) or group by week 1-run interactions (F = 0.02, *p* = 0.899).

### Serum Biomarkers

A total of 48 participants (WP = 23, CHO = 25) were included in the analysis of serum testosterone. The ANOVA was conducted on the log transformed testosterone data due to violation of the assumption of normality of residuals. There was a significant main effect of time (F = 13.74, *p* < 0.01), however, there was no main effect of group (F = 0.89, *p* = 0.35) or group by time interactions (F = 0, *p* = 0.95). A total of 47 participants (WP = 23, CHO = 25) were included in the analysis of serum cortisol. There was a significant main effect of time (F = 4.38, p = 0.04), however, there was no main effect of group (F = 0.34, *p* = 0.56) or group by time interactions (F = 1.88, *p* = 0.18). A total of 48 participants (WP = 23, CHO = 25) were included in the analysis of serum T:C. There was a significant main effect of time (F = 20.15, *p* < 0.01), however, there was no main effect of group (F = 0.75, *p* = 0.39) or group by time interactions (F = 0.8, *p* = 0.38).

**Figure 3:**
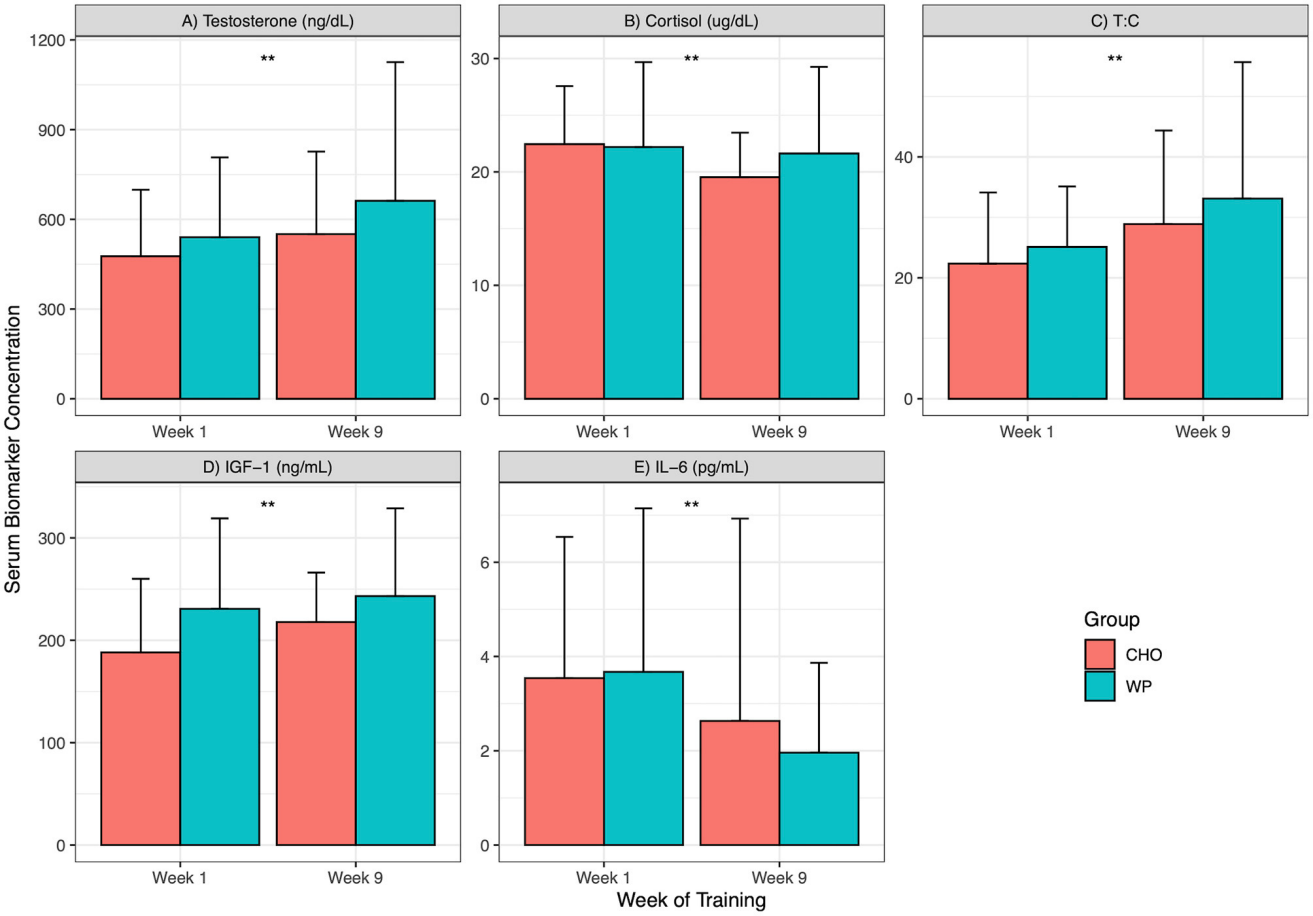
Biomarker response across IET. Biomarkers at weeks 1 and 9. ng/dL, nanogram per deciliter; ug/dL, microgram per deciliter; T:C, Testosterone to Cortisol ratio; ng/mL, nanogram per milliliter; pg/mL, pictograms per milliliter; CHO, Carbohydrate supplement group; WP, Whey protein supplement group; Data presented as mean ± standard deviation. **indicates significant main effect of time (Week 1 vs. Week 9) for both groups; ++indicates a significant group by time interaction. Panels: **(A)** Testosterone; **(B)** Cortisol; **(C)** Testosterone to Cortisol Ratio; **(D)** Insulin-Like Growth Factor 1; **(E)** Interleukin 6.

A total of 48 participants (WP = 23, CHO = 25) were included in the analysis of serum IGF-1. There was a significant main effect of time (F = 8.07, *p* < 0.01), however, there was no main effect of group (F = 2.81, *p* = 0.1) or group by time interactions (F = 1.30, *p* = 0.26). Lastly, we investigated the effects of supplementation on IL-6, a marker of inflammation. A total of 36 participants (WP = 17, CHO = 19) were included in the analysis of serum IL-6. Due to violation of the assumption of normality of residuals, the ANOVA was conducted on the log transformed IL-6 data. There was a significant main effect of time (F = 17.92, *p* < 0.01), however, there was no main effect of group (F = 0.02, *p* = 0.9) or group by time interactions (F = 0.15, *p* = 0.71). The biomarker responses are summarized in Figure 3.

## Discussion

This project examined if 8 weeks of a single daily serving of WP compared to CHO supplementation influenced physical performance, blood biomarkers, and body composition across IET. Our primary findings were: (1) WP was related to a significant reduction in FM during IET; (2) WP had differential effects on FFM depending on the soldiers' FFM upon entry into IET; (3) there was no statistically significant benefit between supplements for physical performance or the anabolic or inflammatory biomarker response to IET. Two important secondary findings were that soldiers increased dietary intake from meals across IET and that training volume was higher in the initial phases of IET in comparison to the later phases. Below we discuss these findings and how these findings (with once-daily WP supplementation) relate to our findings using twice daily supplementation with WP daily in the same population.

Soldiers consuming WP daily had a significant reduction in FM during IET. The WP group lost an additional 1.2 kg of FM with a larger effect size than CHO (WP = −0.24, CHO = −0.1). There were no significant differences in overall caloric intake or training volume completed between groups. Thus, the losses in FM seen here were likely not influenced by those variables. This is similar to our previous work in IET soldiers that found a significant reduction in FM in the WP group that consumed 2 servings (80 g total) of WP daily. IET soldiers consuming 2 servings per day lost an additional 1.8 kg of FM in comparison to the group consuming two CHO servings per day. The potential impact of WP on FM agrees with studies in non-military populations as well (40, 41). WP has been shown to promote FM loss in conjunction with exercise in healthy (41, 42) and obese individuals (40, 41). Animal and cell culture models suggest WP may promote fat metabolism by influencing both adipose and muscle (43, 44). WP has been shown to impact adipose tissue by upregulating signaling pathways associated with the breakdown of triglycerides into FFA (43, 44), fat oxidation (45), thermogenesis (43), and antagonists to fatty acid synthesis (44). Conversely, in myotubes, WP has been shown to upregulate signaling pathways related to free fatty acid transport to mitochondria (43) and mitochondrial ability to oxidize free fatty acids (44). Additionally, myotubes cultured in serum from humans who consumed WP in the fasted state had improved GLUT4 translocation which would promote uptake of glucose to the muscle (46). Thus, WP may contribute to FM reductions by promoting the breakdown of adipose, suppressing the synthesis of FFA, and improving transport and oxidation of free fatty acids in both adipose and muscle tissue. Another potential way WP can impact FM is the thermic effect of food. Previous work has shown that protein has a higher thermic effect than both carbohydrate and fat intake (47). WP has been shown to increase the thermic effect of food (48) and to a greater extent than other protein sources such as soy and casein (49). Therefore, WP supplementation may be a viable option for IET soldiers and individuals who are engaging in exercise training while trying to reduce FM.

WP was also found to be more beneficial for FFM in individuals who had higher FFM at week 1 relative to soldiers with lower FFM and those in the CHO group. This is evidenced by the significant group by baseline FFM interaction. We then used the multivariate model to predict what a soldier's week 9 FFM would be if the individual were in the WP vs. the CHO group at different baseline FFM (5, 10 kg above or below average). In summary, an individual who has higher FFM at week 1 would have a higher predicted week 9 FFM if he were in the WP vs. the CHO group. Interestingly, we followed this up with correlational analysis and found that body weight was significantly, inversely correlated with relative protein intakes at weeks one (R^2^ = −0.66, *p* < 0.001) and nine (R^2^ = −0.56, *p* < 0.001). Further exploration showed that when IET soldiers were binned into groups based on baseline BM, only those where week one BM five kg or more below average consumed 2 or more g/kg/day of protein. Interestingly individuals 10 kg above average week one BM consumed only 1.7 g/kg/day of protein. Organizational recommendations and systematic reviews of the literature suggest that daily protein intakes should be between 1.6 and 2 (50) and 1.7 and 2.2 g/kg/day (51). Therefore, individuals with lower BM in our cohort were able to consume protein intakes on the higher end of the recommended ranges whereas individuals with higher BM were closer to the lower end of this range. These daily requirements may increase to 2–3 g/kg/day in individuals who train in energy-restricted conditions (52), such as may occur in IET (3). Collectively, this suggests that individuals with higher BM or FFM entering into IET may benefit from additional supplementation to help elevate protein intake to optimal levels to optimize the FFM response to training.

The overall group-by-time interaction for FFM was not significant (*p* = 0.07). However, the low *p*-value considered in light of that the WP group gained on average 1.2 kg of FFM vs. only 0.1 kg in CHO and had a larger effect size (WP = 0.1, CHO = 0), suggests that WP may have a clinically relevant effect on FFM in IET soldiers. The lack of statistical significance in the current work may be due to the large response heterogeneity in the cohort. The change in FFM was 0.1 on average with a standard deviation of 3 kg in CHO and 1.2 with a standard deviation of 2.4 kg in the WP group. One potential driver of the variability in response in the CHO group is that protein intake from diet alone was adequate to maximize the FFM response to IET. Previous work in British IET, suggests that nitrogen balance can be attained, at least in the initial weeks, by consumption of 1.5 g/kg/day of protein intake (53). Additionally, a meta-analysis summarizing the literature on supplementation in strenuous military environments suggested protein intakes between 1.7 and 2.2 g/kg/day are recommended (51). Here we report that the CHO group consumed on average 2.2 g/kg/day. It is also possible that additional caloric intake from supplementation, in general, may be beneficial for FFM response to IET. Previously we reported that 90% of IET soldiers gained FFM when consuming two supplement servings per day (21). Overall, in this study, approximately 69% of all IET soldiers gained FFM when consuming one supplement serving per day. Other work reported only 36% of male soldiers gained FFM when no supplementation is given during IET (5). Thus, it is possible there is a dose-response benefit of additional energy intake during IET to combat the negative energy balance that has been previously reported during IET training (3). However, this distinction cannot be made in the current study due to the lack of direct comparison of one vs. two servings with a non-supplemented control group. Future research needs to expand our work by comparing WP and CHO at various doses with a non-supplemented control group.

The body composition presented here should be interpreted with caution, due to the method used. Single-frequency bioelectrical impedance analysis of body composition, as used in the current investigation, has been reported to be a valid and reliable method for assessing body composition (54–56), but may under/over-predict FM and FFM (55, 57). Thus, caution should be used when drawing conclusions about the precise characterization of body composition of IET soldiers from the current investigation. However, the body composition responses to IET and supplementation presented in the current investigation should be considered reliable as controls were in place to optimize the reliability of the results. Estimation equation (54) and conditions prior to assessment (58) can impact the accuracy of SF BIA results. Here we used the Lukaski equation, which has been shown to be a valid estimator of FFM in comparison to hydrostatic underwater weighing and Dual X-ray Absorptiometry (DXA) (56, 57). To address the influence of conditions prior to assessment, we performed body composition measures at the same time of day (early morning), prior to exercise, in the fasted, hydrated state, all of which may impact body composition assessments in SF-BIA (58) and non-SF-BIA methods such as DXA (59, 60). Additionally, we aimed to minimize the influence of electrode placement by having the same team member perform electrode placement on all soldiers. Specifically, the body composition device used in this investigation has reliability in relation to FFM and FM as measured by DXA in obese and athletic populations but may underestimate FM and overestimate FFM (55, 57).

Both the WP and CHO groups improved in overall performance during IET training. This was expected as the physical fitness program is designed to take untrained civilians and make them into trained tactical athletes. The lack of difference in endurance between the groups may be explained in the similar levels of carbohydrate consumption. Current recommendations for athletes involved in moderate, high, and very high volumes of exercise are 5–7 g/kg/day, 6–10 g/kg/day, and 8–12g/kg/day respectively to restore muscle glycogen stores that fuel endurance exercise (61, 62). IET soldiers experience training volumes in the high to very high range but are consuming carbohydrate intakes that are below or at best, on the lower range of the recommendations for their physical activity levels regardless of supplement groups (3, 4, 61, 62). Additionally, there was only a 1.1 g/kg/day difference in carbohydrate intake between groups. Previous work has shown that a difference of 2 g/kg/day of carbohydrate showed no difference in pro or macro-glycogen (subfractions of muscle glycogen that are responsive to diet), only the combined total muscle glycogen levels (63). Therefore, the 1.1 g/kg/day difference between groups may not have been large enough to elevate muscle glycogen stores to a level that would lead to substantial differences in endurance performance. Dietary intakes may also have contributed to the lack of statistical difference in push-up performance. Although there was a significant group difference at the end of IET, both groups increased relative protein intakes to at least 2.2 (WP: 2.5, CHO: 2.2) g/kg/day. Protein intakes at this level are at or above the upper amounts of the current recommended protein intakes for military populations and may have been adequate to support the strength adaptations in IET soldiers (51). Another possible contributor to this is the large variability in individual response. On the group level, there was a large effect size of WP (Cohen's D: 0.52) and a medium effect size of CHO (Cohens D: 0.35) and the WP group gained on average four more push-ups relative to the CHO group. This is similar to what we have previously reported (21). However, the standard deviation of the mean difference was thirteen for CHO with an average improvement of eight push-ups and nine for WP with an average improvement of twelve push-ups. Large variability in the response to IET has been shown elsewhere revealing very large improvements (over 100% improvement) to even losses in push-up performance across training (64). The large variability along with the knowledge that we were only able to obtain physical performance data from three out of the four platoons, may have contributed to the lack of statistical significance in physical performance. Regarding the large variability in response across IET, future work would be highly impactful that is designed to explore the factors that contribute to the response variability so that the IET soldier's response to IET can be optimized.

Serum IGF-1, testosterone, and the T:C ratio significantly increased, whereas IL-6 decreased regardless of the supplementation group across the 8 weeks of IET. Physiologically, IGF-1 and testosterone play important roles in stimulating muscle protein synthesis (65, 66) and enhancing satellite cell activity to increase the myonuclear number and enhance hypertrophy (67, 68). Conversely, cortisol has catabolic effects on skeletal muscle (69) and its increase relative to the concentration of testosterone (T:C) has been related to decreases in performance in athletic environments (13). Studies in similar IET environments outside of the United States consistently show decreases in IGF-1 and increases in Cortisol. The testosterone response is more heterogeneous. One study showed an increase, another shows no change, whereas another shows an increase in initial weeks (1–4) followed by a decrease in the final weeks (5–7). Studies in US Army Ranger training have reported that IGF-1 and testosterone decrease in response to large volumes of training and inadequate energy intake (8, 70). Here, we report that regardless of supplement group, IGF-1 increased, and cortisol decreased, both of which the opposite typically occurs in military training environments. The biomarker decrease in previous studies was thought to reflect an imbalance between training volume and nutritional intake. This imbalance can be restored by increased nutritional intake (12). Therefore, it is possible that additional nutritional intake by supplementation, in general, is beneficial for the biomarker response to IET. However, this statement is limited in that the base, typical hormonal response to IET is not adequately characterized. More work is needed to establish the typical hormonal response to IET in United States IET environments.

We also report that IL-6 decreased across IET. IL-6 is released post-exercise and plays a variety of roles, one of which is stimulating the inflammatory response to muscle damage (71). Chronic elevations in IL-6 have been linked to overtraining (20). Previous work in Israeli IET revealed there was no statistically significant change in IL-6 in male (72) and female (72, 73) recruits across 4 months of training. Another study in Australian IET reported no change in IL-6 across 8 weeks of IET (74). We did not replicate these findings in the current investigation. One potential factor was that IET soldiers in our cohort consumed high levels of protein from their diet. One study in marathon runners showed that while supplementation with soy protein did not have an effect, individuals who consumed higher dietary protein (over 20% of daily caloric intake from protein) had a reduced IL-6 and overall inflammatory response to a marathon (75). Studies on the acute effect of protein supplementation vary in protein dose, type, and results as some report a reduction in IL-6 post-exercise (76) while others report no effect and suggest that meeting energy intake needs may be more important (77). These are all important considerations for the current results as they lend potential explanations for the IL-6 response observed here. Overall participants: consumed on average 19% (±2%) of daily calories from protein at week 9 and received additional caloric intake via calorie-matched supplements. Furthermore, participants for both groups had access to dietary protein in the post-exercise period as physical fitness training for Army IET soldiers occurs early in the morning and is followed by breakfast. It is also important to note that IL-6 also plays a key role in stimulating the immune response to pathogens (16) and is elevated by psychological stress (78). Therefore, it is possible that week 1 levels of IL-6 could have been elevated at pre-intervention due to immunizations, close exposure to a new group of people coming from diverse locations, or stress. This would create an artificial elevation in IL-6 at week 1 and appear to be a reduction in IL-6 across IET. Overall, similar to the hormonal response to IET, the inflammatory response to Army IET is not well characterized and more work needs to be done to characterize the typical inflammatory response of IET soldiers. Considering the collective catabolic (cortisol), inflammatory (IL-6), and anabolic (testosterone, IGF-1) hormonal response observed here, the physiologic environment seems to be one that is beneficial for optimal response to IET.

Although secondary, there were two interesting findings regarding diet and physical activity. Of concern was that supplementation would not be additional nutrition to the soldier's diet but would instead lead to decreased caloric consumption during meals. To address this, we collected diet logs before implementation of supplementation during the baseline week, allowing us to compare dietary intake from the dining facility alone at baseline to see if there was an increase in food consumption across IET. Here, as in our past investigation (21), we report that IET soldiers increased dietary intake from meals consumed from the dining facility and that both groups in the current investigation increased absolute and relative (relative to body weight) macronutrient intake across IET. It is important to note that this was not a primary aim of this investigation and, therefore, future research needs to be conducted to determine if supplementation does negatively impact food consumption. Another secondary important finding from this study is that physical activity (i.e., training volume) was significantly different across the IET phase. The red phase (first 3 weeks of IET) was significantly higher in time spent in light activity than all other phases and on average had the highest total training volume. This is in agreement with our previous work that found training volume was higher during the initial weeks of training (3, 4). Reports from the Center for Disease Control (CDC) suggest that <30% of individuals in the U.S. aged 18–35 participate in 300 min per week of moderate or 150 min of vigorous-intensity exercise (79). Here, we report that IET soldiers participate in over 400 min per day of at least light intensity exercise. Thus, IET soldiers may experience rapid increases in training volume as they perform more physical activity in 1 day than much of the US population performs in 1 week. US Army training command has been working to resolve these issues.

What should finally be noted is how the current dataset relates to our previous study where IET Soldiers were provided two servings of WP vs. a calorie-matched CHO supplement (21). The current investigation was conducted in a separate cohort, with different IET cadre, military occupation specialty, and training, with only one serving of WP or CHO once per day. The mean differences (WP minus CHO) for FFM were 1.1 (single serving) and 0.6 (two servings) kg higher in the WP vs. CHO groups. WP decreased FM 1.2 (single serving) and 1.8 (two servings) kg more on average and improved push-up performance on average about 4.3 (single servings) and 4.2 (two servings), relative to the change in the CHO group. Though not all of these were determined to be statistically significant, the consistency of these results, even though IET cohort and leadership were different, suggest that WP may benefit body composition changes and strength endurance during IET. More information on comparisons between our prior and current studies can be found in Table 5.

**Table 5:** Consistency of between group mean differences across cohorts.

		**Single WP/CHO serving**	**Two WP/CHO servings**
**Variable**	**Group**	**Mean Difference [CI]**	**Mean Difference [CI]**
FFM (kg)	CHO	0.1 [−5.8, 5.9]	3.6 [2.3, 4.9]
	WP	1.2 [−3.5, 5.8]	4.2 [3.1, 5.4]
	Diff	1.1	0.6
FM (kg)	CHO	−0.9 [−5.9, 4.1]	−2.7 [−4.0, −1.3]
	WP	−2.1 [−7.8, 3.6]	−4.5 [−5.8, −3.2]
	Diff	−1.2	−1.8
PU (reps)	CHO	7.8 [−17.3, 32.9]	2.6 [−0.7, 6.0]
	WP	12.1 [−5.8, 30.1]	6.8 [2.9, 10.7]
	Diff	4.3	4.2

*Mean difference: the average difference across IET with 95% CIs at weeks 1 and 9 for FFM and FM and weeks 2 and 8 for PU. Diff, Mean Difference in WP minus Mean Difference in CHO. FFM, Fat-Free Mass; FM, Fat Mass; PU, Push-ups completed in 1 min; WP, Whey protein supplement group; CHO, Carbohydrate supplement group*.

There are limitations to this study. One limitation in this study was that performance data was obtained from 75% of the participants. This resulted in more data being collected for the WP group in comparison to the CHO. We were still able to obtain 55 (WP = 37, CHO = 17) data points. Another limitation is that performance data was collected by multiple testers. While inter-rater reliability could influence the findings of this study, it is notable that drill sergeants administered all tests and are highly trained in conducting the APFT. They administer the test often and IET soldier graduation is dependent upon the APFT. Caution should be taken regarding the body composition results (critiqued in detail above). While the reliability of a single frequency has been established previously (54–56), the characterization of true FFM and FM may not be as precise as other methods such as underwater weighing and DXA (55, 57). Regarding the analysis of biomarkers, it is notable that blood draws were collected only at the week 1 and week 9-time points due to limited access to the soldiers during the IET period. Ideally, more sampling time points would be completed to better describe the typical hormonal and inflammatory response of soldiers to Army IET environments, which is not well characterized. Another limitation is the timing of supplement consumption. We were able to record adherence to consuming supplementation but were not able to gather adherence as to the time of consumption/dispersion of supplements being before bed. Drill sergeants were asked to disperse and IET soldiers were instructed to consume supplements before bed, but the research team was not present due to trying to be minimally invasive into the IET training schedule. Finally, our discussion of the potential influence of supplement dose (one vs. two servings) must be considered in the context that we did not perform a direct comparison in this investigation. Our work investigating two servings per day was completed previously in a different IET cohort (21).

## Conclusion

Once-daily supplementation with WP significantly decreased FM and enhanced gains in FFM in individuals who entered IET with higher FFM compared to those with lower FFM. The consistency of mean changes and effect sizes in FM and FFM and previous cohorts of Army IET suggest that WP may be beneficial for soldiers' body composition response during IET. However, there was no significant influence of WP on physical performance or biomarkers of the physiologic response to IET. The lack of response may be due to high relative dietary protein intakes in IET soldiers in the current cohort or may suggest that more than one serving is needed to optimize performance.

## Data Availability Statement

The datasets presented in this article are not readily available because of Army data sharing restrictions. They may be available upon special arrangement. Requests to access the datasets should be directed to jms0018@auburn.edu.

## Ethics Statement

The studies involving human participants were reviewed and approved by the Auburn University Institutional Review Board, and the Director, Research and Analysis Directorate Army Center. The patients/participants provided their written informed consent to participate in this study.

## Author Contributions

JM, JS, KY, KRL, and MDR: conceptualization. JM, JS, DB, PM, KY, KRL, MDR, and KDL: methodology. JM, CH, PM, and KRL: software/analysis. JM, JS, CH, PM, PR, KY, KRL, and MDR: validation. JM, PM, KY, and KRL: formal analysis. JS, JM, KDL, DB, CH, MAR, PM, PR, KY, KRL, and MDR: investigation. JS and MDR: resources. JS, JM, KDL, DB, CH, MAR, PM, PR, KY, and MDR: data collection and quality assurance. JM, JS, and MDR: writing-original draft preparation and funding acquisition. JS, KDL, DB, CH, MAR, PM, PR, KY, KRL, and MDR: writing-review and editing. JM and MDR: visualization. JS and JM: supervision. JS, JM, KDL, and MDR: project coordination/administration. All authors contributed to the article and approved the submitted version.

## Funding

Funding for data collection and conduction of this study were provided by the Warrior Research Center. Both WP and CHO supplements were donated to the Warrior Research Center by Bionutritional Research Group (Irvine, CA, USA) and Hilmar Ingredients (Hilmar, CA, USA). Dr. Chris Lockwood of Lockwood, LLC (C.M.L., Draper, UT, USA) solicited these donations and provided additional financial support in ensuring that products were formulated, tested for content, and blinded accordingly.

## Conflict of Interest

Supplements were donated to the Warrior Research Center by Bionutritional Research Group (Irvine, CA, USA) and Hilmar Ingredients (Hilmar, CA, USA). Dr. Chris Lockwood of Lockwood, LLC (C.M.L., Draper, UT, USA) solicited these donations. CH is employed by Fitomics, LLC. The data presented here are honestly presented without falsification, fabrication, or inappropriate manipulation. The remaining authors declare that the research was conducted in the absence of any commercial or financial relationships that could be construed as a potential conflict of interest.

## Publisher's Note

All claims expressed in this article are solely those of the authors and do not necessarily represent those of their affiliated organizations, or those of the publisher, the editors and the reviewers. Any product that may be evaluated in this article, or claim that may be made by its manufacturer, is not guaranteed or endorsed by the publisher.
